# A Discrete Brain Storm Optimization Algorithm for Hybrid Flowshop Scheduling Problems with Batch Production at Last Stage in the Steelmaking-Refining-Continuous Casting Process

**DOI:** 10.3390/s24227137

**Published:** 2024-11-06

**Authors:** Kunkun Peng, Chunjiang Zhang, Weiming Shen, Xinfu Pang, Yanlan Mei, Xudong Deng

**Affiliations:** 1 School of Management, Wuhan University of Science and Technology, Wuhan 430065, China; pengkunkun@wust.edu.cn (K.P.); meiyanlan@wust.edu.cn (Y.M.); dengxudong@wust.edu.cn (X.D.); 2 State Key Laboratory of Intelligent Manufacturing Equipment and Technology, Huazhong University of Science and Technology, Wuhan 430074, China; zhangcj@hust.edu.cn; 3 School of Mechanical & Electrical Engineering, Kunming University, Kunming 650214, China; 4 School of Automation, Shenyang Institute of Engineering, Shenyang 110136, China; pangxf@sie.edu.cn

**Keywords:** steelmaking-refining-continuous casting, iron and steel, energy saving, hybrid flowshop scheduling, batch production, brain storm optimization

## Abstract

The iron and steel industry is energy-intensive due to the large volume of steel produced and its high-temperature and high-weight characteristics, sensors such as high-temperature application sensors can be utilized to collect production data and support the process control and optimization. Steelmaking-refining-continuous casting (SRCC) is a bottleneck in the iron and steel production process. SRCC scheduling problems are worldwide problems and NP-hard. The problems are not only important for iron and steel enterprises to enhance production efficiency, but also play a significant role in saving energy and reducing resource consumption. SRCC scheduling problems can be modeled as hybrid flowshop scheduling problems with batch production at the last stage. In this paper, a Discrete Brain Storm Optimization (DBSO) algorithm is proposed to handle SRCC scheduling problems. In the proposed DBSO, population initialization and cluster center replacement are specially designed to enhance the intensification abilities. Moreover, a perturbation operator is devised to enhance its diversification abilities. Furthermore, a new individual generation operator is devised to improve the intensification and diversification abilities simultaneously. Experimental results have demonstrated that the proposed DBSO is an efficient method for solving SRCC scheduling problems.

## 1. Introduction

The iron and steel industry is the foundation of the global economy [1]. It is energy-intensive in nature since it has a large volume of steel produced, and has high-temperature and high-weight characteristics [2,3]. Sensors such as high-temperature application sensors and rolling testing sensors are fundamental data resources and executors utilized for the process control and optimization of the smart steel manufacturing [4]. The iron and steel production process is a complex technological process, where a number of jobs have to be processed and several kinds of machines are utilized. Thus, production scheduling is very important for the modern iron and steel industry [5]. Steelmaking-refining-continuous casting (SRCC) is the critical part and bottleneck of the iron and steel production process [2,6]. The production scheduling problems in the SRCC process are named SRCC scheduling problems. SRCC scheduling will help to enhance production efficiency. Pang et al. [7] indicated that in real-world applications, for problems with an average number of 66 charges, compared with manual schedules, the average completion times of schedules obtained by effective SRCC scheduling methods were reduced by 9.96%. This is highly meaningful for saving energy and reducing resource consumption. Therefore, SRCC scheduling problems are significant for the iron and steel industry. However, the problems are NP-hard and recognized as some of the most difficult industrial scheduling problems [8,9]. Moreover, due to the large volume of steel produced, the problems are large-scale, and thus difficult to be solved.

Due to the significance and complexity of SRCC scheduling problems, many researchers have investigated the problems, and a series of mathematical models and scheduling methods have been developed. In the existing studies, many researchers modeled SRCC scheduling problems as hybrid flowshop scheduling problems with batch production at the last stage [10,11], where the batch production at the last stage is a constraint that the charges belonging to the same batch should be consecutively processed at the last stage. Among the existing scheduling methods, Lagrangian relaxation-based methods [12,13] and metaheuristics-based methods are very popular.

With respect to the metaheuristics-based methods, different kinds of metaheuristics have been utilized by a number of scholars to deal with the problems, because metaheuristics have several advantages: (1) Metaheuristics have been demonstrated to be very effective for solving NP-hard problems. (2) Metaheuristics are relatively easy to understand and implement. Here, we list some of the recent metaheuristics-based SCC scheduling methods. For instance, the authors of [9,11,14] developed different Artificial Bee Colony (ABC) algorithms for SRCC scheduling problems. Peng et al. [15] presented an improved ABC for SRCC scheduling problems under dynamic environments. Li et al. [16] designed an improved Fruit Fly Optimization Algorithm (FOA), while Li, Pan, and Mao [17] further designed an improved FOA to solve SRCC scheduling problems under dynamic environments, and very recently, Peng et al. [18] tailored variable neighborhood descent to handle similar problems. Moreover, there are also research taking into account other features of the SRCC production process; for example, Peng et al. [19] developed a Grey Wolf Optimizer (GWO) for SRCC scheduling problems considering adjustable processing times, and Long et al. [20] proposed an improved Genetic Algorithm (GA) for SRCC scheduling problems considering adjustable processing times and stage skipping. Lu and Qiao [10] designed an adaptive GA to solve SRCC scheduling problems with the objective of minimizing extra energy consumption. Tang and Wang [21] designed a Particle Swarm Optimization (PSO) algorithm for SRCC scheduling problems with the objective of minimizing total weighted completion time. For more information, readers can refer to two recent reviews by [22,23].

The Brain Storm Optimization (BSO) algorithm is a recent and promising metaheuristic, which was developed by Shi in 2011 [24]. It has also been successfully applied to handle a series of complicated shop scheduling problems such as energy-efficient stochastic hybrid open shop multi-objective scheduling problems [25], multi-objective energy-efficient distributed assembly no-wait flow shop scheduling problem [26], integrated multi-constraint open shop scheduling and vehicle routing [27], and integrated distributed flow shop scheduling and distribution problems with time windows [28]. These studies have clearly demonstrated that BSO is an effective metaheuristic to deal with such complex shop scheduling problems.

To the best of our knowledge, there is no work on employing BSO to solve SRCC scheduling problems until now. BSO was inspired by the brainstorming process, which has been successfully used to construct ideas to solve complex problems [24,29], while most metaheuristics for SRCC scheduling problems were inspired by collective behaviors of animals. BSO should be superior to these metaheuristics since human beings are the most intelligent animals in the world [24]. In this paper, we explore how to utilize BSO to handle SRCC scheduling problems, which belong to complex shop scheduling problems. That is, we try to design an effective BSO algorithm for the problems. The canonical BSO algorithm cannot be directly utilized, since it was originally proposed for continuous optimization problems. The main contributions of this paper are as follows:

(1) The Discrete Brain Storm Optimization (DSBO) algorithm is specially proposed for SRCC scheduling problems. To the best of our knowledge, this is the first attempt to utilize BSO to solve the problems.
(2) In the proposed DBSO, four key algorithm components, i.e., population initialization, cluster center replacement, new individual generation, and perturbation, are delicately designed to improve its intensification and diversification abilities. These components collectively enhance the search power of the proposed DBSO greatly.

The rest of this paper is structured in the following way. Section 2 illustrates SRCC scheduling problems in detail. Section 3 describes traditional BSO. Section 4 describes the components and framework of the presented DBSO, while Section 5 reports the experimental comparison results. Finally, the conclusions are given in Section 6.

## 2. SRCC Scheduling Problems

### 2.1. Problem Description

**Figure 1:**
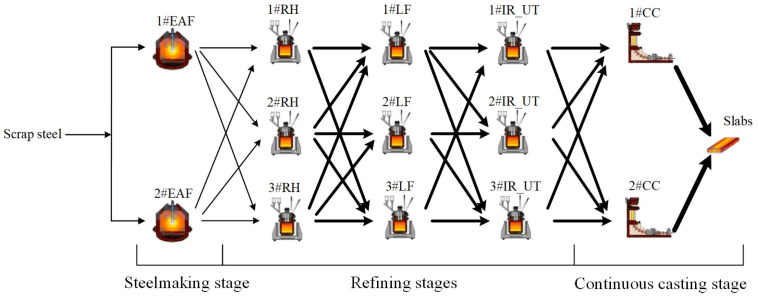
Illustration of a simple SRCC process [15,18].

The SRCC process includes three kinds of production stages: steelmaking, refining, and continuous casting, and the three stages are implemented successively. The SRCC process studied in this paper is Electric Arc Furnace (EAF)-based, where the EAF is the processing machine for the steelmaking stage. In the steelmaking stage, scrap steel is processed in an EAF to reduce unnecessary impurities. A full ladle load of grade-specific molten steel is named a charge. When the steelmaking stage is finished, the charge is transferred into a refining furnace to further eliminate unwanted impurities and add required alloy components. In this stage, the refining furnace is the processing machine, for example, Ladle Furnace (LF) and RH-vacuum degassing (RH). The production stage is named the refining stage. Note that different steels have different production routes, and thus, for some steels, the number of the refining stages may be one, two, three, or four. In the steelmaking and refining stages, the charge is a basic production unit. Subsequently, the continuous casting stage is executed, and a set of charges are consecutively processed in the same tundish of a continuous caster. The set of charges are named a cast and the continuous caster is the processing machine. A cast is a basic production unit of this stage. In this paper, it is assumed that the processing sequence of the charges in a cast is given at the upper planning level in advance [10,11,30]. Figure 1 illustrates a simple SRCC process, which includes steelmaking, three refining, and continuous casting stages.

The SRCC scheduling problems investigated in this paper can be described as follows. Given a number of charges to be processed, the production sequence and machine assignments of these charges in the continuous casting stage have been determined at the upper planning level, and the goal of SRCC scheduling problems is to determine a schedule with optimal objective values, where the optimization objectives are to minimize sojourn time and the earliness/tardiness of cast starting. The optimal schedule contains the starting times and completion times of each charge at each stage, and the machine assignments of each charge at the steelmaking and refining stages. Meanwhile, the optimal schedule must satisfy a number of complex constraints, e.g., the charges belonging to the same cast must be cast continuously, and a charge must wait if there is no machine available when it arrives at the refining or continuous casting stage [10,11].

### 2.2. Mathematical Model

The mathematical model of SRCC scheduling problems is described as follows [10,11,14].

Indices and parameters:

*i:* Charge index, *i* ∈ *C* = {1, 2, …, *n*}, where *n* denotes the total number of charges.

*j:* Stage index, *j* ∈ *J* = {1, 2, …, *S*}, where *S* denotes the total number of stages.

*k:* Machine index, *k* ∈ *M_i_*, where *M_j_* denotes the machine set of the *j*-th stage.

*J_e_* = {1, 2, …, *S −* 1}: Stage set except the continuous casting stage.

*C = C*_1_∪*C*_2_∪…∪*C_nc_*, where $C_{p_{1}}\cap C_{p_{2}}=\emptyset$, ∀*p*_1_ ≠ *p*_2_ ∈ *NC* = {1, 2, …, *nc*}, and *nc* denotes the total number of casts.

$CC_{k}=\{C_{a_{k-1}+1},C_{a_{k-1}+2},\ldots ,C_{a_{k}}\}$: Set of casts to be processed on the *k*-th continuous caster, where *a_k−_*_1_ + 1, *a_k−_*_1_ + 2, *a_k_* ∈ *NC*, and *a*_0_ = 0.

*PT_ij_*: Processing time of charge *i* at stage *j.*

*TT_j_*_,*j*+1_: Transferring time from stage *j* to *j* + 1.

*SpT_p_*: Setup time of cast *C_p_*, *p* ∈ *NC.*

*ReT_jk_*: Release time of machine *k* at stage *j.*

*Q*: A very large positive number.

Decision variables:

*ST_ij_*: Starting time of charge *j* at stage *i.*

*x_ijk_*: If charge *i* is assigned to machine *k* at stage *j*, *x_ijk_* = 1; otherwise, *x_ijk_* = 0.

$y_{i_{1}i_{2}j}$: If charge *i*_1_ is preceding charge *i*_2_ to be processed at stage *j*, $y_{i_{1},i_{2},j}=1$; if *i*_1_ and *i*_2_ start at the same time, $y_{i_{1}i_{2}j}=1/2$; otherwise $y_{i_{1}i_{2}j}=0$.

(1) $$\min F=\sum_{i=1}^{3}c_{i}F_{i}$$

(2) $$F_{1}=\frac{\sum_{i=1}^{n}\left(ST_{iS}-ST_{i1}-PT_{i1}\right)}{n}$$

(3) $$F_{2}=\sum_{p=1}^{nc}\max(DD_{p}-ST_{a_{p-1}+1,S},0)$$

(4) $$F_{3}=\sum_{p=1}^{nc}\max(ST_{a_{p-1}+1,S}-DD_{p},0)$$

s.t.

(5) $$\sum_{k\in M_{j}}x_{ijk}=1,\forall i\in C,\;j\in J$$

(6) $$ST_{ij}-x_{ijk}ReT_{jk}\geq 0,\forall i\in C,j\in J_{e},k\in M_{j}$$

(7) $$y_{i_{1}i_{2}j}+y_{i_{2}i_{1}j}=1,\forall \;i_{1},i_{2}\in C,j\in J_{e}$$

(8) $$ST_{i_{2}j}-(ST_{i_{1}j}+PT_{i_{1}j})+Q(3-y_{i_{1}i_{2}j}-x_{i_{1}jk}-x_{i_{2}jk})\geq 0,\forall \;i_{1},i_{2}\in C，k\in M_{j},j\in J_{e}$$

(9) $$ST_{i,j+1}-(ST_{ij}+PT_{ij}+TT_{j,j+1})\geq 0,\forall i\in C,j\in J_{e}$$

(10) $$ST_{iS}-x_{iSk}ReT_{Sk}-SpT_{a_{k-1}+1}\geq 0,i=C_{a_{k-1}}+1\;\wedge \;i\in C,k\in M_{S}$$

(11) $$ST_{iS}+PT_{iS}=ST_{i+1,S},\forall \;i,i+1\in C_{p},p\in NC$$

(12) $$ST_{a_{p}+1,S}\geq ST_{a_{p}S}+PT_{a_{p}S}+SpT_{p+1},\forall C_{p},C_{p+1}\in CC_{k}$$

(13) $$x_{ijk}\in \left\{0,1\right\},i\in C,j\in J,k\in M_{j}$$

(14) $$y_{i_{1}i_{2}j}\in \left\{0,1/2,1\right\},i_{1},\;i_{2}\in C,j\in J$$

Formula (1) is to minimize the weighed sum of the sojourn time and earliness/tardiness of cast starting, where the sojourn time, earliness of cast starting, and tardiness of cast starting are illustrated by Formulas (2), (3), and (4), respectively. Formulas (5)–(9) are charge-related constraints while Formulas (10)–(13) are cast-related constraints. Formula (6) ensures that each charge must be processed in all the stages, where a charge must be processed by exactly one machine at each stage. Formula (6) emphasizes that for the steelmaking and refining stages, the starting time of a charge on a machine must be no smaller than the release time of the machine. Formulas (7) and (8) make sure that, in the steelmaking and refining stages, for two charges processed on the same machine, the second charge cannot be processed before the first one is finished. Formula (9) ensures that for each charge’s two consecutive operations, the second one cannot be processed before the first one is finished and transferred to the machine corresponding to the second one. Formula (10) emphasizes the condition that the first cast on a continuous caster can be processed. Formula (11) is the casting constraint for the charges in a cast, i.e., the charges in a cast must be cast continuously. Formula (12) ensures that for a continuous caster’s two consecutive casts, the second one cannot be processed before the first one is finished and the subsequent setup operation is finished. Formulas (13) and (14) state the value ranges of *x_ijk_* and $y_{i_{1}i_{2}j}$.

## 3. Introduction of Canonical Brain Storm Optimization (BSO) Algorithm

The canonical BSO algorithm starts with an initial population generated randomly (i.e., population initialization), and then iteratively executes a search process of solution clustering–cluster center replacement–new individual generation, until the termination condition is reached.

Let *t* be the iteration number, *T* be the maximum number of iterations, *P_r_* be the probability of replacing a selected cluster center with a new individual, *P_og_* be the probability of selecting one cluster to generate new individuals, *P_s_* be the probability of selecting a cluster center, *P_c_* be the probability of selecting a cluster center to generate a new individual, and *P_tc_* be the probability of selecting two cluster centers to generate a new individual. The canonical BSO algorithm (Algorithm 1) is illustrated as follows [24,27,28].
**Algorithm 1.** Framework of the Canonical BSOStep 1.**Parameter setting.** Set *t* = 1, individual index *m* = 1, and solution set *SS* = *Ø*.Step 2.**Population Initialization**. Randomly generate an initial population Pop_1_ including *M* individuals.Step 3.**Solution clustering**. Cluster the individuals in *Pop_t_* into *c* clusters. For each cluster, evaluate the individuals, rank them according to corresponding fitness values, and record the best one as the cluster center.Step 4.**Cluster center replacement.** Randomly select a cluster center with probability P_r_, and replace it with an individual generated randomly.Step 5.**New individual generation.** Generate a random real number *rd* ∈ [0, 1]. If *rd* < *P_og_*, go to Step 5.1; otherwise, go to Step 5.2.
Step 5.1.Randomly select a cluster with probability *P_s_*. Generate a random real number *rd_1_* ∈ [0, 1]. If *rd*_1_ < *P_c_*, select the cluster center and add a random value to it to generate a new individual; otherwise, randomly select an individual from the cluster and add a random value to it to generate a new individual. Go to Step 5.3.Step 5.2.Randomly select two clusters. Generate a random real number *rd*_2_ ∈ [0, 1]. If *rd*_2_ < *P_tc_*, the two cluster centers are combined and then added with a random value to generate a new individual; otherwise, two individuals from each selected cluster are randomly selected to be combined and added with a random value to generate a new individual. Go to Step 5.3.Step 5.3.If the newly generated individual *NC_m_* is compared with *Pop_tm_*, the better one is kept and recorded as the new individual.Step 6.Set *m* = *m* + l. If *m* ≤ *M*, go to Step 5. Otherwise, set *m* = 1, and go to Step 7.Step 7.Set *t* = *t* + l. If the termination condition has been met (e.g., *t* > *T*), return the best solution found by BSO; otherwise, go to Step 3.

## 4. The Proposed Discrete Brain Storm Optimization (DBSO)

The proposed DBSO is described in this section. First of all, encoding and decoding methods are illustrated. Then, other key elements of DBSO are described sequentially, i.e., population initialization, solution clustering, cluster center replacement, new individual generation, selection, and perturbation. In the end, the framework of DBSO is given.

### 4.1. Encoding and Decoding

The permutation of all the charges is utilized as the encoding, which is the same as that in [14].

The decoding process transforms an individual into an SRCC schedule. The decoding method used in this paper is the same as that in [14]. More specifically, the decoding method includes two parts, i.e., forward decoding and backward decoding. The forward decoding process generates a schedule which can finish all the charges as soon as possible. This is achieved by allocating the charges to the first available machine at each stage. However, the resulting schedule may be not feasible, since the continuous casting constraint is not considered in the forward decoding. Thus, backward decoding is further utilized to make it feasible. More specifically, for an individual (say *Pop_tk_*), the decoding can be stated as follows, where Steps 1–3 and Steps 4–6 correspond to the forward and backward decoding, respectively.

Step 1. At the steelmaking stage, select the charges from the encoding corresponding to *Pop_tk_*, where the charges are selected from beginning to end. For each selected charge, allocate it to the first available EAF, calculate the completion time of the charge, and then update the available time of the EAF.

Step 2. At the refining stages, from the first refining stage to the last one, select the charges from the encoding corresponding to *Pop_tk_*, where the charges are also selected from beginning to end. For each selected charge, allocate it to the first available refining furnace, calculate the completion time of the charge, and then update the available time of the refining furnace.

Step 3. At the continuous casting stage, for each cast in each continuous caster, process the charges in the cast one by one. Note that the latter charge can be processed only when the former charge has been finished. Moreover, for the second cast to the last cast in each continuous caster, the starting time of the first charge in the cast is set to be the sum of the completion time of the former cast and the setup time for the cast.

Step 4. At the continuous casting stage, for each cast in each continuous caster, from the second last charge to the first charge, set the completion time of the charge to be the starting time of the latter charge, and update the starting time of the charge.

Step 5. At the refining stages, from the last refining stage to the first one, for the charges in each refining furnace, from the last charge to the first one, update the completion time of the charge by shifting the charges right.

Step 6. At the steelmaking stage, from the last EAF to the first one, for the charges in each EAF, from the last charge to the first one, update the completion time of the charge by shifting the charges right.

### 4.2. Population Initialization

An initial population with certain intensification and diversification may help to enhance the search performance of the proposed DBSO. To achieve this goal, some initial individuals of a certain quality are generated by heuristics while others are generated randomly. Note that in this paper, the term ‘individual’ is the same as the term ‘solution’. The initial population (say *Pop*_1_) is constructed by the following strategy.

Step 1. For each cast, according to its planned starting time, calculate the starting time of each charge in the cast at the continuous casting stage.

Step 2. For all the charges, sort them in the non-decreasing order of the starting times. Then, a charge permutation is obtained, denoted by *Pop*_11_, i.e., the first individual of *Pop*_1_.

Step 3. Execute the following operation several times (say *g* times) to construct *g* individuals: generate a copy solution of *Pop*_11_, and then implement the Swap operator to it, where the Swap operator means exchanging two charges at two positions selected randomly.

Step 4. Execute the following operation *g* times to construct *g* individuals: generate a copy solution of *Pop*_11_, and then implement the Pairwise Swap operator to it, where the Pairwise Swap operator means exchanging two charges at adjacent positions selected randomly.

Step 5. Execute the following operation *g* times to construct *g* individuals: generate a copy solution of *Pop*_11_, and then implement the Insert operator to it, where the Insert operator consists of removing a charge at a position and then reinserting it into another position.

Step 6. Generate the remaining *NP*-3*g*-1 individuals randomly, where *NP* denotes the population size of the presented DBSO.

### 4.3. Solution Clustering

The solution clustering method in [28] is employed in this paper to cluster the individuals in *Pop_t_* into *m* clusters. Let *q* be the number of individuals in a cluster, where *q* = *N*/*m*, and let *i* be the cluster index, set *i* = 1. The solution clustering can be stated as the following.

Step 1. Generate a copy of *Pop_t_*, denoted as *Pop_copy_*.

Step 2. For the individuals in *Pop_copy_*, evaluate and sort them in the non-decreasing order of the objective function values.

Step 3. Select the best *m* individuals from *Pop_copy_*, set them as *m* cluster centers, and then remove them from *Pop_copy_*.

Step 4. Select the best *q −* 1 individuals from *Pop_copy_*, allocate them to the *i*-th cluster, and then remove them from *Pop_copy_*.

Step 5. Set *i* = *i* + 1. If *i* > *m*, go to Step 6; otherwise, go to Step 4.

Step 6. *m* clusters are constructed, where each cluster has a cluster center and *q −* 1 individuals.

### 4.4. Cluster Center Replacement

In the cluster center replacement of the traditional BSO, a cluster center is selected randomly, and further replaced by an individual generated randomly. However, the selected cluster center is one of the best individuals in the population, it contains some useful information. In contrast, an individual generated randomly is generally much worse. Replacing the cluster center with such an individual may influence the quality of the individuals obtained by the following new individual generation operator. Therefore, in this paper, the cluster center selected randomly is replaced by another cluster center selected randomly, which contains more useful information than an individual generated randomly. The cluster center replacement can be described as follows.

Step 1. Generate a random real number *rand* ∈ [0, 1]. If *rand* < *P_r_*, go to Step 2; otherwise, stop.

Step 2. Randomly select a cluster center (say *CC*_1_). Then, further randomly select a cluster center from the remaining cluster centers, say *CC*_2_.

Step 3. Exchange the two cluster centers *CC*_1_ and *CC*_2_.

### 4.5. New Individual Generation

The generation of new individuals involves two different generation operators, where an individual or two individuals are employed to generate new individuals. More specifically, the two different generation operators can be described as follows: (1) For the case of utilizing an individual, Multiswap and control-based local search are applied. (2) For the case of utilizing two individuals, the PMX-based operator is applied. The two generation operators are implemented randomly, where the first- and second-generation operators are implemented with probabilities *P_og_* and 1 − *P_og_*, respectively.

#### 4.5.1. Multiswap and Control-Based Local Search

In the Multiswap and control-based local search, the Multiswap operator [11] is used for the randomly selected cluster center or individual to generate new solutions. The Multiswap operator is a neighboring operator designed by Pan et al. [11], and it includes several (say *q*) Pairwise Swap operations, where *q* is an integer generated randomly in a given range. If the newly obtained solutions are better than the current solution, they will be accepted as the current solution; otherwise, if the newly obtained solutions are slightly worse than the current solution, they will also be accepted as the current solution. This idea is derived from [31]. Let *Iter_max_* be the maximum number of iterations, and the Multiswap and control local search are illustrated as follows.

Step 1. Set iteration number *iter* = 1.

Step 2. Randomly select a cluster.

Step 3. Generate a real number *rand* ∈ [0, 1] randomly. If *rand* < *P_s_*, in the selected cluster, select the cluster center, and go to Step 5; otherwise, go to Step 4.

Step 4. In the selected cluster, select an individual randomly, and go to Step 5.

Step 5. Set the current solution as the selected cluster center or individual.

Step 6. Apply the Multiswap operator to the current solution. If the obtained solution is better than the local optimal solution (say *X_L_*), update *X_L_*. If the obtained solution is better or slightly worse than the current solution (say *X_C_*), i.e., *F*(*X_L_*) ≤ *R × F* (*X_C_*), where *R* is set to be 1.005 in this paper, utilize the obtained solution to update the current solution.

Step 7. Set *iter* = *iter* + 1. If *iter* > *Iter_max_*, go to Step 8; otherwise, go to Step 6.

Step 8. Return *X_L_* as the new individual generated by the Multiswap and control based local search.

#### 4.5.2. PMX-Based Operator

In the PMX-based operator, PMX is applied to two solutions for generating new solutions, because PMX is known to be an efficient crossover operator for solving shop scheduling problems. More information about PMX can be found in [32,33]. Moreover, unlike the new individual generation of traditional BSO, the two solutions are two cluster centers selected randomly or two individuals selected randomly from two clusters in the PMX-based operator, and the two solutions are obtained as follows: (1) Two cluster centers are selected randomly from the cluster centers as two individuals for PMX. (2) Randomly select two cluster centers, and for each cluster, randomly select two individuals from the cluster, and the better one is utilized as an individual for PMX. (3) A cluster center is selected randomly from the cluster centers as an individual for PMX. Then, randomly select another cluster center, randomly select two individuals from the cluster, and the better one is utilized as an individual for PMX. This will enhance the diversification abilities of the proposed DBSO. More specifically, the PMX-based operator is illustrated as follows.

Step 1. Randomly select two clusters, say *C*_1_ and *C*_2_.

Step 2. For new individual generation, select two solutions from the two clusters using Steps 2.1–2.5.

Step 2.1. Generate a real number *rand*_1_ ∈ [0, 1] randomly. If *rand*_1_ < *P_tc_*, the cluster centers of *C*_1_ and *C*_2_ are employed as the two solutions, and go to Step 3; otherwise, go to Step 2.2.

Step 2.2. Generate a real number *rand*_2_ ∈ [0, 1] randomly. If 0 ≤ *rand*_2_ < 1/3, go to Step 2.3; if 1/3 ≤ *rand*_2_ < 2/3, go to Step 2.4; if 2/3 ≤ *rand*_2_ ≤ 1, go to Step 2.5.

Step 2.3. Randomly select two individuals from *C*_1_, and find the better one of the two. Meanwhile, randomly select two individuals from *C*_2_, and find the better one of the two. Employ the two selected individuals as the two solutions.

Step 2.4. Randomly select two individuals from *C*_2_, and find the better one of the two. Employ it and the cluster center of *C*_1_ as the two solutions.

Step 2.5. Randomly select two individuals from *C*_1_, and find the better one of the two. Employ it and the cluster center of *C*_2_ as the two solutions.

Step 3. Implement PMX to the above selected two solutions to generate two new solutions.

Step 4. Implement the Multiswap and control-based local search to the two obtained individuals.

Step 5. Return the obtained individuals in Step 4 as individuals generated by the PMX-based operator.

### 4.6. Selection

During new individual generation, one or two new individuals are generated, and then one should decide whether they will be retained or removed. In traditional BSO, if the newly generated individual (say *NC_n_*) is better than the individual with the same index (say *Pop_tn_*), update *Pop_tn_* by *NC_n_*. This may mean discarding some ‘good’ solutions. A selection operator is employed for selecting better solutions to construct the new population. More specifically, the selection operator in [28] is illustrated as follows.

Step 1. Store the new individuals obtained by executing the new individual generation operator.

Step 2. Merge these solutions with the current population, and sort them in the non-decreasing order of their objective function values.

Step 3. Select the top *N* individuals from the merged solution set, and set them as the current population.

### 4.7. Perturbation

In this paper, a perturbation operator is designed to enhance the diversification abilities of the proposed DBSO. This is achieved by exploring different but promising searching space by mutating the individuals in the population with a certain probability. More precisely, the perturbation operator is illustrated as follows.

Step 1. Set individual index *i* = 1.

Step 2. For the *i*-th individual in the current population, i.e., *Pop_ti_*, randomly generate a real number *rd*_1_ ∈ [0, 1]. If *rd*_1_ < *P_m_*, set *j* = 1, and go to Step 3; otherwise, go to Step 5.

Step 3. Randomly generate a real number *rd*_2_ ∈ [0, 1]. If *rd*_2_ < *P_mto_*, apply the Insert operator to *Pop_ti_*; otherwise, apply the Multiswap operator to *Pop_ti_*.

Step 4. Set *j* = *j* + 1. If *j* > 3, go to Step 5; otherwise, go to Step 3.

Step 5. Set *i* = *i* + 1. If *i* > *N*, stop; otherwise, go to Step 2.

### 4.8. Framework of the Proposed DBSO

**Figure 2:**
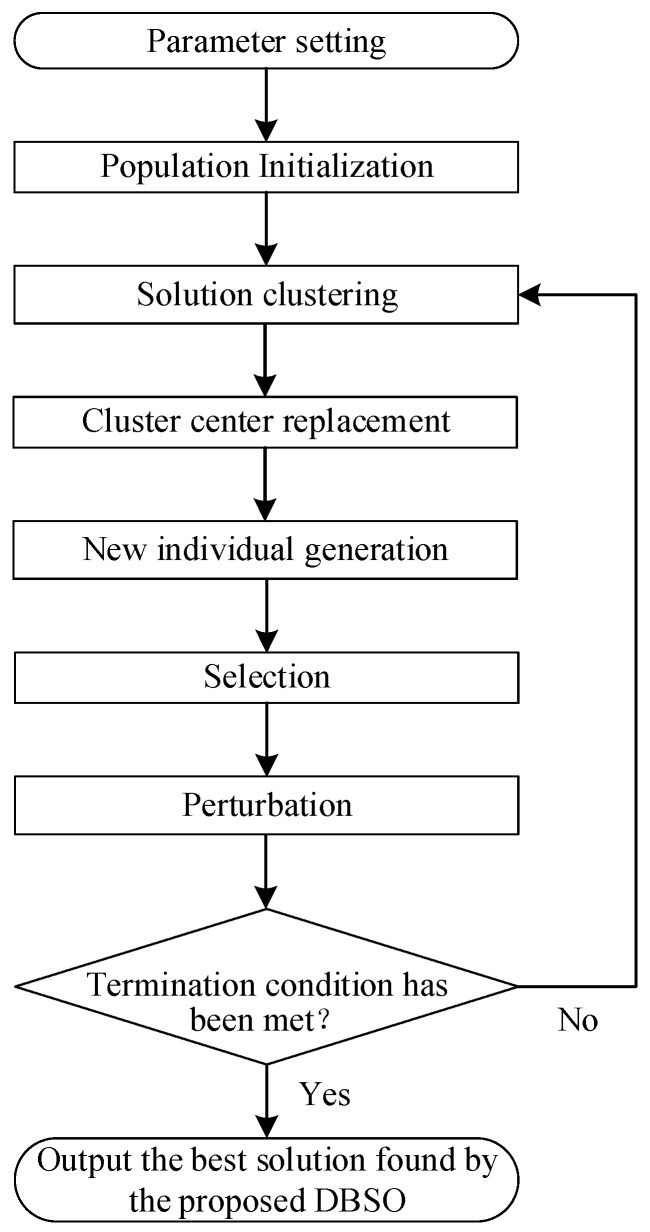
The flowchart of the proposed DBSO.

Let *gBest* be the best solution found so far, *t* be the iteration number, *T* be the maximum number of iterations, *P_r_* be the probability of replacing a selected cluster center with a new individual, *P_og_* be the probability of selecting one cluster to generate new individuals, *P_s_* be the probability of selecting a cluster center, *P_c_* be the probability of selecting the selected cluster center to generate a new individual, and *P_tc_* be the probability of selecting two cluster centers to generate a new individual. Figure 2 shows the flowchart of the proposed DBSO. More specifically, the framework of DBSO is given as follows. The computational complexity of the population initialization is *O* (*n* × *MN* × *NP*), where *MN* denotes the total number of machines. The computational complexity of the new individual generation is *O* (*n* × *MN* × *NP* × *MI*), where *MI* denotes the maximum iteration number. Therefore, the computational complexity of DBSO is not large. When the scales of the problems are larger, DBSO can also deal with the problems within acceptable computational times. Furthermore, as can be seen from Figure 2 and the framework of DBSO, DBSO is enhanced by incorporating improved population initialization, improved cluster center replacement, improved new individual generation, and perturbation, and we believe that DBSO can solve the problems with larger scale effectively (Algorithm 2).
**Algorithm 2.** Framework of the proposed DBSOStep 1.**Parameter setting.** Set *t* = 1, and individual index *m* = 1.Step 2.**Population Initialization**. Generate an initial population *Pop_t_* by the strategy illustrated in Section 4.2. Find the best solution in *Pop_t_* (say *Pop_tk_*), and set *gBest* = *Pop_tk_*.Step 3.**Solution clustering**. Evaluate the individuals in *Pop_t_* and sort them in the non-decreasing order of the objective function value. Select the best *c* individuals as the cluster centers, and allocate the remaining individuals to the *c* cluster centers.Step 4.**Cluster center replacement.** Randomly select and exchange two cluster centers with probability *P_r_*.Step 5.**New individual generation.** Generate a random real number *rd* ∈ [0, 1]. If *rd* < *P_og_*_,_ go to Step 5.1; otherwise, go to Step 5.2.
Step 5.1.Randomly select a cluster with probability *P_s_*. Randomly generate a real number *rd*_1_ ∈ [0, 1]. If *rd*_1_ < *P_c_*, select the cluster center; otherwise, randomly select an individual from the cluster. For the selected cluster center or individual, implement the Multiswap and control-based local search in Section 4.5.1 to generate a new individual. Go to Step 5.4.Step 5.2.Randomly select two clusters. Randomly generate a real number *rd*_2_ ∈ [0, 1]. If *rd*_2_ < *P_tc_*, implement PMX to the two cluster centers of the selected two clusters to generate two new individuals, further implement the Multiswap and control-based local search to the two individuals, and go to Step 5.4; otherwise, go to Step 5.3.Step 5.3.Randomly generate a real number *rd*_3_ ∈ [0, 1]. If 0 ≤ *rd*_3_ < 1*/3*, for each selected cluster, randomly select two individuals, and find the better one of the two; if 1/3 ≤ *rd*_3_ < 2/3, for the second cluster, randomly select two individuals, and find the better one of the two. Then, select the cluster center for the first cluster; if 2/3 ≤ *rand*_3_ ≤ 1, for the first cluster, randomly select two individuals, and find the better one of the two. Then, select the cluster center for the second cluster. Implement PMX to the selected two individuals to generate two new individuals, and further implement the Multiswap and control-based local search to the two obtained individuals. Go to Step 5.4.Step 5.4.Add the newly generated individual(s) to a solution archive; if the newly generated individual(s) is better than *gBest*, update *gBest*.Step 6.Set *m* = *m* + l. If *m* ≤ *M*, go to Step 5; otherwise, set *m* = 1, and go to Step 7.Step 7.**Selection.** Add the individuals in the current population to the solution archive, select the best *NP* individuals from the solution archive, and set them as *Pop_t_*. If the best solution of *Pop_t_* is better than *gBest*, update *gBest*.Step 8.**Perturbation.** For each individual in *Pop_t_*, implement the perturbation stated in Section 4.7; if the obtained solution is better than *gBest*, update *gBest*.Step 9.Set *t* = *t*+l. If the termination condition has been met (e.g., *t* > *T* or the maximum execution time is reached or the maximum iteration number *MI* is reached), return *gBest* as the best solution found by the proposed DBSO; otherwise, go to Step 3.

## 5. Experimental Results

The proposed DBSO is a metaheuristics-based method, which cannot ensure the optimal solution; therefore, comparison experiments were conducted to verify its efficiency. More specifically, we compared it with six algorithms on 30 instances. The six compared algorithms include the following: (1) five versions of BSO, (2) an effective GA proposed by [33] for hybrid flowshop problems with sequence-dependent setup times and machine eligibility, denoted as GA_R_. The algorithms were coded in C++ and executed on a computer with 2.30 GHz CPU and 16 GB RAM using the Windows 10 operation system. The following Section 5.2 and Section 5.2 will describe the 30 instances and the specific experimental results, respectively.

### 5.1. Instance Description

By referring to Lu and Qiao [10] and Pan et al. [11], 30 test instances were randomly generated as follows. In each instance, the corresponding SRCC production process contained the steelmaking, refining, and continuous casting stages, where the number of the refining stages was one. The three stages had three EAFs, five refining furnaces, and four continuous casters, respectively, where the release time of each machine was zero. The processing times of each charge in the steelmaking, refining, and continuous casting stages were generated randomly in the intervals [30, 42], [37, 50], and [38, 44], respectively. The transfer times between the steelmaking and refining stages and the transfer times between the refining stage and continuous casting stages were generated randomly in the interval [10,15]. In the continuous casting stage, each continuous caster processed three or four casts. For each cast, the setup time was 100, and the number of contained charges was generated randomly in the interval [8,12].

### 5.2. Comparisons Analysis

Detailed comparison results between the proposed DBSO and the above-mentioned six algorithms are given in this section. The details of the six compared algorithms are stated as follows:

(1) For the five versions of BSO (denoted as BSO_M_, BSO_S_, BSO_P_, BSO_I_, and BSO_IV_), during population initialization, an individual was generated as the first individual, as described in Section 4.2, while the other individuals were generated randomly. The solution clustering was the same as described in Section 4.3. In the cluster center replacement, a cluster center selected randomly was replaced by an individual generated randomly. In the new individual generation, for the case of utilizing two individuals to generate new individuals, the PMX crossover operator was employed to generate two new individuals; if the better one of the two new individuals was better than the individual in the population, the corresponding individual in the population was updated. For the case of utilizing an individual to generate new individuals, different methods were employed; if the obtained individual was better than the individual in the population, the corresponding individual in the population was updated. In BSO_M_, BSO_S_, BSO_P_, BSO_I_, and BSO_IV_, the Multiswap operator, Swap operator, Pairwise exchange operator, Insert operator, and Inversion operator, were employed to create a new individual.
(2) For GA_R_, the population initialization was the same as the above five versions of BSO, i.e., an individual was generated as the first individual, as described in Section 4.2, while the other individuals were generated randomly.

The maximum elapsed CPU time limit was employed in this study as the termination condition. In this study, three termination conditions were employed, i.e., elapsed CPU time limit of 10, 20, and 30 s. The reasons for utilizing the three elapsed CPU time limits were two-fold: (1) practical situations require a schedule in 30 s [11]; (2) more comparison experiments can be executed to test the performance of the proposed DBSO. For each instance, each algorithm was run ten times independently. Each run would obtain an objective function value. Then, the average value of the ten objective function values was calculated, and was further utilized to calculate Relative Percentage Increase (RPI). RPI was employed to measure the algorithm performance, denoted as *RPI_Alg_*, i.e., the RPI of algorithm *Alg*, and it was computed by the following formula.

(15) $$RPI_{Alg}=\frac{F_{Alg}-F_{cb}}{F_{cb}}\times 100\%$$

where *F_Alg_* denotes the average result of algorithm *Alg*, while *F_cb_* denotes the best value among the current algorithms.

In regard to the parameters of DBSO, for simplicity, in this paper we did not fine-tune the parameters *P_r_*, *P_og_*, *P_s_*, *P_c_*, and *P_tc_*, and set them the same as those in the original BSO [24]. Note that since DBSO is a metaheuristics-based method, the parameters influence the performance of DBSO, and perhaps better results may be obtained if these parameters were fine-tuned. Moreover, the maximum number of iterations of the Multiswap and control-based local search *Iter_max_* was set to be 30. With respect to the parameters of BSO_M_, BSO_S_, BSO_P_, BSO_I_, and BSO_IV_, the parameters *P_r_*, *P_og_*, *P_s_*, *P_c_*, and *P_tc_* were also set to be the same as those in the original BSO [24]. For the parameters of GA_R_, the parameters in [34] were employed.

Table 1 lists the comparison results under the elapsed CPU time limit of 10 s. In the table, the first column lists the instance number, other columns describe the RPIs of different algorithms, and the last column represents the average RPI of the 30 instances. It can be observed from Table 1 that, under a CPU time of 10 s, DBSO performed considerably better than BSO_M_, BSO_S_, BSO_P_, BSO_I_, BSO_IV_, and GA_R_. More specifically, the average RPI of DBSO was 0.01%, while the average RPIs of BSO_M_, BSO_S_, BSO_P_, BSO_I_, BSO_IV_, and GA_R_ were 3.43%, 11.45%, 9.47%, 11.39%, 12.22%, and 19.76%, respectively.

Table 2 presents the experimental results under an elapsed CPU time limit of 20 s. It can be seen from Table 2 that DBSO also significantly outperformed the other six compared algorithms. For all the instances, the average RPIs of DBSO were all 0, while the corresponding average RPIs of BSO_M_, BSO_S_, BSO_P_, BSO_I_, BSO_IV_, and GA_R_ were 3.80%, 12.06%, 9.89%, 11.92%, 12.96%, and 18.47%, respectively.

Table 3 reports the experimental results under an elapsed CPU time limit of 30 s. It can be seen from Table 3 that DBSO also performed the best among these algorithms. The specific average RPIs were similar to those results under an elapsed CPU time limit of 10 and 20 s. More specifically, the average RPIs of DBSO was 0, while the corresponding average RPIs of BSO_M_, BSO_S_, BSO_P_, BSO_I_, BSO_IV_, and GA_R_ were 3.79%, 12.32%, 9.99%, 12.12%, 13.27%, and 17.66%, respectively. In brief, the proposed DBSO outperformed the GA_R_ and the five versions of BSO. Meanwhile, the five versions of BSO all performed better than GA_R_.

Moreover, to demonstrate the effectiveness of two important algorithm components of DBSO, i.e., new individual generation and perturbation, we compared DBSO with its variant (denoted as DBSO_V_). In DBSO_V_, for the new individual generation, when utilizing two individuals to generate new individuals, the PMX crossover operator was employed to generate two new individuals. For the case of utilizing an individual to generate new individuals, the Swap operator was employed. The other components of DBSO_V_ were the same as those of DBSO. The average RPIs of DBSO and DBSO_V_ are listed in Table 4. According to Table 4, one can observe that, under the elapsed CPU time limits of 10, 20, and 30 s, the average RPIs of DBSO were all 0, while those of DBSO_V_ were 11.30%, 11.90%, and 12.20%, respectively. That is, DBSO performed better than DBSO_V_.

Furthermore, DBSO was employed to solve a case, and the Gantt chart corresponding to the best schedule obtained by DBSO was also given to better show the scheduling result. The case included 22 charges to be processed sequentially in three stages: a steelmaking stage, a refining stage, and a continuous casting stage. The three stages had three Electric Arc Furnaces, five refining furnaces, and four continuous casters, respectively. Each continuous caster processed a cast. That is, there were four casts, where the first cast contained charges 1–6, the second cast contained charges 7–12, the third cast contained charges 13–17, and the fourth cast contained charges 18–22.

**Figure 3:**
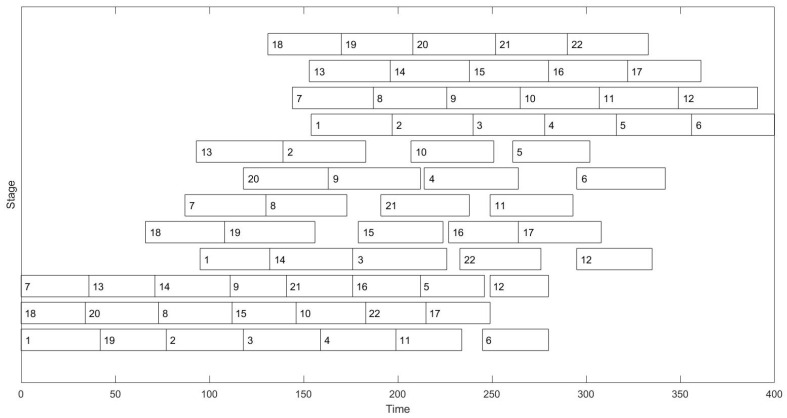
The Gantt chart of the best schedule obtained by DBSO.

The processing times of the 22 charges are described in Table 5, specifically. In Table 5, the first row gives the charge number, while the first column describes the stage number. Moreover, the second, third, and fourth rows describe the processing times of these charges in the steelmaking, refining, and continuous casting stages. The best schedule obtained by DBSO achieved an objective function value of 897.73, where the sojourn time, earliness of cast starting, and tardiness of cast starting were 84.773, 20, and 3, respectively. The Gantt chart corresponding to the schedule is given in Figure 3. From Figure 3, one can observe that charges 1–6 belonging to the first cast were processed continuously in the continuous casting stage. The situations were the same for charges 7–12, charges 13–17, and charges 18–22.

In conclusion, DBSO is an efficient approach for solving SRCC scheduling problems. We believe that the reasons why DBSO performs better are as follows: the population initialization, cluster center replacement, new individual generation, and perturbation collectively enhance the search efficiency of DBSO greatly. With these efficient components, the performance of DBSO is enhanced greatly and utilized to handle problems on a larger scale.

## 6. Conclusions

Following the successful works on adopting BSO to solve several shop scheduling problems, this paper investigated an effective DBSO algorithm for SRCC scheduling problems. To the best of our knowledge, this is the first work on tailoring BSO to deal with the problems. In the presented DBSO, the population initialization, cluster center replacement, new individual generation operator, and perturbation operator are elaborately devised to improve the search power. The cluster center replacement exchanges the cluster centers with a certain probability, which will help to enhance the diversification abilities of DBSO. The new individual generation operator is devised with six generation options, which will also improve the diversification abilities. Moreover, the Multiswap and control-based local search is embedded into the new individual generation operator, which enhances the intensification abilities. The perturbation operator generates new solutions with a certain probability, which will also improve the diversification abilities, and thus help DBSO escape the local optima. Comparison experiments showed that DBSO performed better than the compared algorithms. This demonstrates that DBSO is an efficient method for handling SRCC scheduling problems.

With regards to the future research, we will carry out the following three aspects: (1) tailor the proposed DBSO to solve other shop scheduling problems [35,36,37], (2) enhance the search performance of the proposed DBSO by incorporating deep learning technologies or hybridizing it with some latest metaheuristics [38,39,40,41], (3) incorporate IoT technologies and sensors for steel manufacturing environments, which results in a big data environment [42]. Such circumstances are more complicated, and DBSO cannot deal with these; therefore, it is necessary to further investigate effective multi-objective scheduling methods [43,44] or dynamic scheduling methods.

## Figures and Tables

**Table 1:** Performance of DBSO in comparison with six compared algorithms with CPU time 10 s.

Instance	DBSO	BSO_M_	BSO_S_	BSO_P_	BSO_I_	BSO_IV_	GA_R_
1	0.00	3.40	11.74	12.57	10.88	11.69	13.55
2	0.03	0.00	9.40	10.08	14.49	16.03	25.75
3	0.00	5.21	7.27	5.49	10.46	6.92	12.29
4	0.00	3.94	10.70	6.67	10.51	10.89	17.66
5	0.00	2.14	8.50	3.64	7.22	8.41	15.39
6	0.00	3.95	11.62	7.81	12.23	14.18	16.36
7	0.00	1.07	10.53	5.67	8.43	10.90	18.50
8	0.00	1.04	2.87	2.24	4.18	3.27	10.52
9	0.00	3.57	9.38	6.52	6.50	7.92	11.77
10	0.00	13.94	25.98	33.25	18.33	27.52	42.82
11	0.00	1.39	5.41	5.18	5.39	5.29	5.68
12	0.00	1.80	16.62	10.71	14.07	13.21	23.86
13	0.00	5.13	21.70	22.27	25.13	28.05	37.27
14	0.00	0.28	10.39	9.17	11.74	9.97	22.30
15	0.00	1.52	9.31	3.85	13.08	5.64	27.58
16	0.00	0.63	6.28	4.57	5.51	6.37	11.16
17	0.00	1.29	10.17	6.50	14.40	11.28	26.20
18	0.00	1.48	9.31	8.33	8.80	9.61	9.50
19	0.00	0.06	0.88	6.93	1.10	7.05	10.78
20	0.00	8.31	15.90	12.26	15.22	14.13	22.31
21	0.00	3.24	5.98	5.95	5.83	5.65	7.86
22	0.41	0.00	13.58	19.50	19.84	23.77	32.85
23	0.00	9.37	14.80	13.01	10.08	13.18	17.36
24	0.00	5.94	24.56	12.08	23.79	21.32	28.65
25	0.00	11.50	11.81	13.09	14.34	15.22	19.62
26	0.00	0.53	20.84	2.50	10.28	13.28	27.66
27	0.00	0.51	6.18	4.60	7.22	9.34	34.61
28	0.00	4.88	10.15	8.01	10.04	10.96	13.47
29	0.00	4.62	15.47	14.92	17.18	18.58	18.37
30	0.00	2.17	6.30	6.67	5.40	6.91	11.11
Average	0.01	3.43	11.45	9.47	11.39	12.22	19.76

**Table 2:** Performance of DBSO in comparison with six compared algorithms with CPU time 20 s.

Instance	DBSO	BSO_M_	BSO_S_	BSO_P_	BSO_I_	BSO_IV_	GA_R_
1	0.00	2.28	12.11	12.72	11.25	12.05	12.88
2	0.00	0.15	8.83	10.40	12.53	15.73	25.05
3	0.00	6.61	9.63	7.70	12.67	9.17	13.43
4	0.00	4.14	10.94	6.06	10.74	11.12	14.53
5	0.00	2.69	8.82	4.23	7.97	9.16	13.97
6	0.00	5.68	13.58	9.16	13.85	16.19	16.84
7	0.00	1.27	11.05	6.00	8.97	11.45	16.01
8	0.00	1.18	3.55	2.88	4.86	3.96	8.23
9	0.00	4.89	10.80	7.80	7.78	9.32	12.57
10	0.00	13.89	24.70	29.62	17.79	27.10	34.34
11	0.00	1.45	5.99	5.64	5.96	5.86	6.15
12	0.00	1.56	17.07	11.02	13.22	13.62	23.45
13	0.00	9.17	27.47	27.98	31.18	33.81	42.00
14	0.00	0.46	10.46	7.43	11.89	10.17	21.56
15	0.00	1.51	9.59	3.84	12.92	5.91	23.21
16	0.00	0.71	6.43	4.43	5.66	6.52	10.14
17	0.00	1.39	10.11	6.78	14.16	11.53	23.76
18	0.00	1.72	9.74	8.43	9.23	10.03	9.67
19	0.00	0.11	1.14	7.08	1.37	7.34	9.97
20	0.00	8.60	16.16	12.67	15.20	14.56	19.50
21	0.00	3.36	4.75	6.14	6.03	5.87	7.17
22	0.00	0.15	13.62	19.67	20.06	24.01	30.92
23	0.00	9.67	15.12	13.33	9.97	13.51	15.85
24	0.00	5.36	24.89	12.33	24.11	21.54	27.77
25	0.00	13.32	13.75	14.92	14.70	17.21	19.77
26	0.00	1.46	22.05	3.49	11.36	14.42	26.61
27	0.00	0.91	6.61	4.30	7.59	9.47	26.64
28	0.00	4.12	9.79	8.51	10.55	11.11	12.98
29	0.00	3.75	16.03	15.47	17.74	19.15	18.77
30	0.00	2.42	7.14	6.72	6.24	7.77	10.33
Average	0.00	3.80	12.06	9.89	11.92	12.96	18.47

**Table 3:** Performance of DBSO in comparison with six compared algorithms with CPU time 30 s.

Instance	DBSO	BSO_M_	BSO_S_	BSO_P_	BSO_I_	BSO_IV_	GA_R_
1	0.00	2.38	12.22	12.77	11.36	12.16	12.49
2	0.00	0.18	8.91	10.27	12.57	15.82	23.89
3	0.00	5.92	9.79	7.86	12.94	9.44	12.76
4	0.00	4.22	11.03	6.06	10.57	11.21	13.87
5	0.00	2.80	8.97	3.82	8.10	9.31	12.84
6	0.00	5.83	12.78	9.14	14.01	16.36	16.38
7	0.00	1.04	11.13	5.98	9.05	11.53	15.14
8	0.00	1.20	3.62	2.58	4.93	4.03	6.88
9	0.00	4.55	11.16	7.82	8.13	9.68	12.69
10	0.00	13.03	25.57	30.05	18.68	27.98	33.56
11	0.00	1.51	6.00	5.71	6.03	5.93	6.15
12	0.00	2.19	17.82	10.94	13.63	14.34	24.04
13	0.00	9.55	29.38	29.76	33.04	35.82	42.01
14	0.00	0.60	10.69	7.65	11.10	10.37	21.19
15	0.00	1.38	9.67	3.77	13.01	5.99	18.13
16	0.00	0.90	6.68	4.66	5.90	6.74	9.15
17	0.00	0.07	10.27	6.91	13.65	11.70	22.84
18	0.00	1.66	9.84	8.23	9.32	10.12	9.64
19	0.00	0.19	1.28	6.78	1.51	7.49	9.22
20	0.00	8.67	16.26	12.55	15.25	14.65	18.21
21	0.00	4.49	5.76	7.09	7.22	7.06	7.89
22	0.00	0.31	13.78	19.87	20.25	24.22	29.67
23	0.00	9.78	15.13	13.45	8.85	13.64	14.91
24	0.00	3.41	25.08	11.99	24.30	21.73	27.39
25	0.00	13.99	13.62	15.52	15.39	17.92	19.63
26	0.00	1.66	22.32	3.39	11.59	14.62	25.06
27	0.00	1.04	6.78	4.29	7.75	9.14	22.65
28	0.00	4.67	10.43	8.78	11.20	11.77	13.20
29	0.00	3.83	16.15	15.35	17.87	19.28	18.88
30	0.00	2.61	7.36	6.75	6.45	7.99	9.54
Average	0.00	3.79	12.32	9.99	12.12	13.27	17.66

**Table 4:** Average RPIs of DBSO and DBSO_V_.

Algorithm	10 s	20 s	30 s
DBSO	0	0	0
DBSO_V_	11.30	11.90	12.20

**Table 5:** The processing times of 22 charges.

	1	2	3	4	5	6	7	8	9	10	11
1	42	41	41	40	34	35	36	39	30	37	35
2	37	44	50	50	41	47	43	43	49	44	44
3	43	43	38	38	40	44	43	39	39	42	42
	12	13	14	15	16	17	18	19	20	21	22
1	31	35	40	34	36	34	34	35	39	35	32
2	40	46	44	45	37	44	42	48	45	47	43
3	42	43	42	42	42	39	39	38	44	38	43

## Data Availability

The data presented in this study are available on request from the corresponding author. The data are not publicly available due to privacy.
